# The high protection of a novel chemo-attenuated sporozoite vaccine requires IL-15-mediated liver CD8^+^ tissue-resident memory T-cell responses

**DOI:** 10.1128/mbio.01131-26

**Published:** 2026-09-24

**Authors:** Nie Tan, Chengyu Zhu, Teyang Li, Shubin Su, Shiming Jiao, Yuanli Gao, Yan Ding, Taiping Liu, Wenyue Xu

**Affiliations:** 1 Department of Pathogenic Biology, Army Medical University (Third Military Medical University) 12525, Chongqing, China; 2 Department of Clinical Laboratory, People’s Hospital of Deyang City, Deyang, China; 3 Key Laboratory of Extreme Environmental Medicine, Ministry of Education of China, Chongqing, China; Georgia Institute of Technology, Atlanta, Georgia, USA

**Keywords:** malaria, vaccine, chemo-attenuated sporozoite, CD8^+^ Trm cells, monocytes

## Abstract

Chemo-attenuated sporozoite vaccines (SPZ-CVacs) are considered promising for malaria prevention, but safety concerns persist regarding antimalarial drugs targeting either liver- or blood-stage parasites. In this study, we found that nitroquine (CI-679), used to eliminate malaria blood-stage parasites, effectively rendered sporozoites attenuated by arresting late liver-stage development. A single vaccination with CI-679-attenuated *Plasmodium berghei* (*P. berghei*) sporozoites conferred robust protection against sporozoite challenge in BALB/c mice. Using mouse models with distinct genetic backgrounds, we demonstrated that the robust protection conferred by the CI-679-attenuated vaccine was primarily associated with the frequency of parasite-specific CD8^+^ tissue-resident memory (Trm) cells mediated by IL-15. Notably, the supplement of IL-15/IL-15Ra sushi complex remarkably increased Trm frequency and improved the protective efficacy of CI-679-attenuated vaccine. Our findings characterize a novel SPZ-CVac and provide mechanistic evidence supporting IL-15 as an adjuvant to enhance the efficacy of pre-erythrocytic stage vaccines.

**IMPORTANCE:**

Malaria remains a devastating global disease. Chemo-attenuated sporozoite vaccines show promise, yet safety concerns persist as current drugs target only liver- or blood-stage parasites. Here, we found nitroquine (CI-679) has a potentially favorable safety profile because of its dual liver- and blood-stage activity, and a single vaccination with CI-679-attenuated *P. berghei* sporozoites conferred robust protection against sporozoite challenge in BALB/c mice. Mechanistically, protection was associated with IL-15-mediated parasite-specific CD8^+^ tissue-resident memory (Trm) cells. Notably, the supplement of IL-15/IL-15Ra sushi complex remarkably increased Trm frequency and enhanced protective efficacy. Therefore, we characterize a novel chemo-attenuated sporozoite vaccine and provide mechanistic evidence supporting IL-15 as an adjuvant for pre-erythrocytic stage vaccines.

## INTRODUCTION

Infection begins when sporozoites are deposited in dermal tissue during an infectious *Anopheles* mosquito bite. Then, the sporozoites reach the liver sinusoids through the bloodstream, traverse the endothelium, and productively invade hepatocytes. Within the hepatocytes, they differentiate from trophozoites to schizonts; a single schizont can generate >10^4^ merozoites that egress into circulation and initiate the pathogenic blood stage (1). The absence of symptoms during pre-erythrocytic infection provides a clinically silent window in which to intercept the parasite and prevent downstream pathology. Vaccination that targets the pre-erythrocytic stage therefore pre-empts all downstream parasitemia and clinical disease, offering the most strategic checkpoint for prophylactic malaria vaccine (2).

Recently, the World Health Organization (WHO) has granted approval for two pre-erythrocytic subunit vaccines, RTS,S/AS01 and R21/Matrix-M, for deployment in regions characterized by moderate to high malaria endemicity (3). Although these vaccines have been shown to significantly mitigate morbidity and mortality rates among children, their efficacy in preventing malaria infection is still under evaluation (4). In contrast, whole sporozoite vaccines, such as radiation-attenuated sporozoites (5), genetically attenuated sporozoites (6), and chemo-attenuated sporozoite vaccine (SPZ-CVac) (7, 8), have demonstrated the ability to induce a high level of sterile protection (>90%) against malaria infection (9). Among these, SPZ-CVac is deemed promising due to its robust immunogenicity and the relatively lower number of sporozoites required to achieve sterile protective immunity (8). Traditionally, sporozoites were achieved only through the blood meal of infected mosquitoes, which contradicted the widespread application of whole sporozoite vaccines. However, recent advancements have revealed that infectious *Plasmodium falciparum* (*P. falciparum*) sporozoites can now be produced *in vitro* (10), potentially addressing the challenges of mass supply and enhancing the feasibility of whole sporozoite vaccines in the future.

In addition to sporozoite production, there are safety concerns regarding the current antimalarial drugs used for prophylaxis. Antimalarial medications such as chloroquine, artemisinin, and mefloquine have previously been employed in the context of SPZ-CVac; however, brief exposure to the blood stage is unavoidable (11–13). The emergence and spread of drug-resistant strains of *P. falciparum* further raise safety concerns regarding the use of these antimalarial drugs in this vaccine approach. Consequently, other antimalarial agents, including primaquine (PQ), pyrimethamine, and certain antibiotics that target parasites at the liver stage, have also been explored for use in SPZ-CVac (14, 15). Nevertheless, safety concerns persist, as the risk of breakthrough blood-stage infections cannot be eliminated.

In this study, we found that the antimalarial drug nitroquine (CI-679) not only inhibits the blood-stage of the parasites but also halts the development of the liver stage. A single administration of CI-679 for prophylaxis demonstrated high protective efficacy; however, this efficacy varied among different host-parasite combinations. Notably, the differing protective immunity between C57BL/6 and BALB/c mice against *P. berghei* sporozoites depended on liver-resident tissue-resident memory (Trm) CD8^+^ T cells mediated by IL-15, and the supplement of IL-15/IL-15Ra sushi complex remarkably increased Trm quantity and improved the protective efficacy of CI-679-attenuated vaccine.

## RESULTS

### CI-679 inhibits malaria liver-stage development

**Fig 1:**
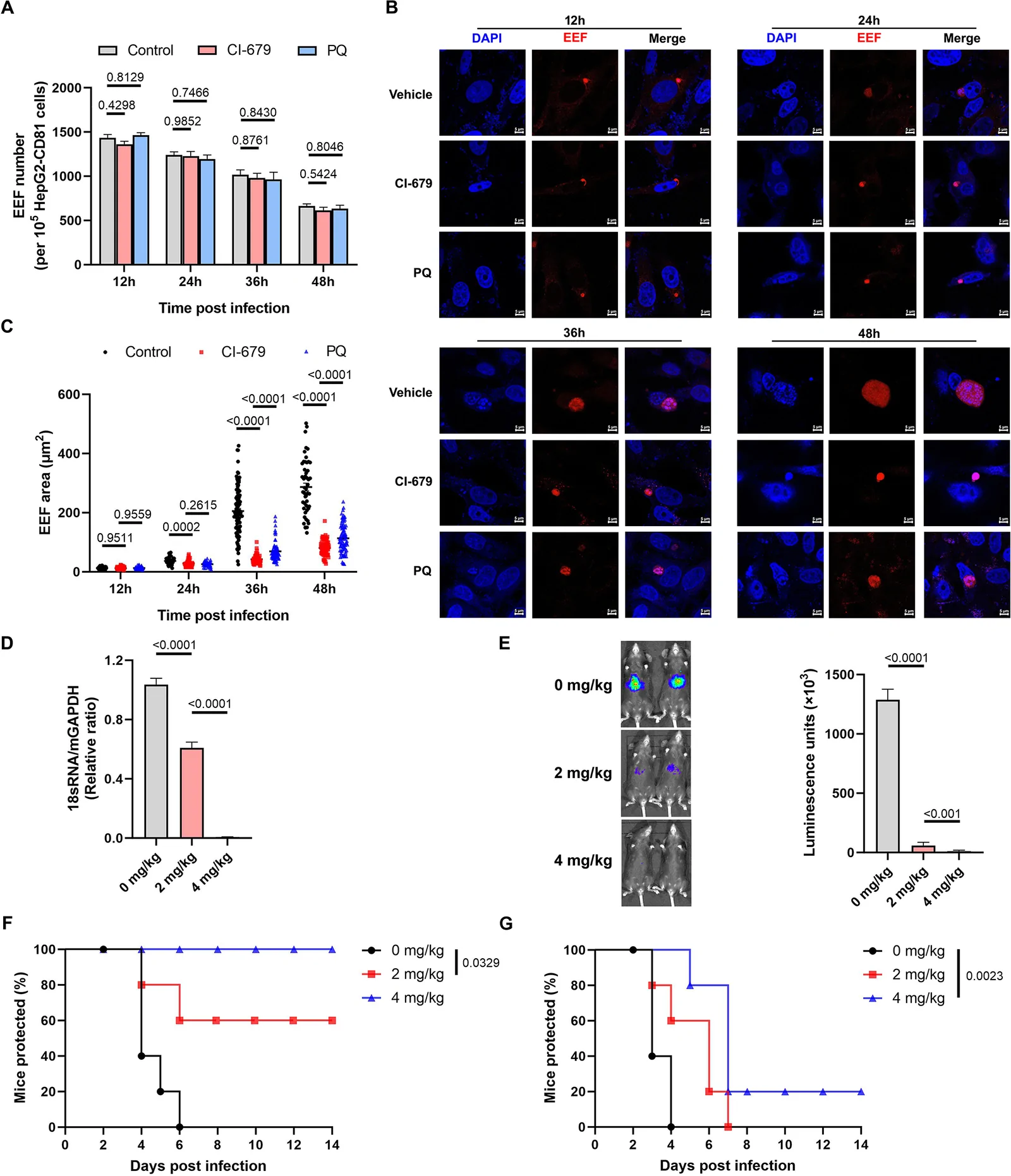
CI-679 inhibits malaria liver-stage development. (**A**) HepG2-CD81 cells were incubated with *P. yoelii* sporozoites for 12, 24, 36, and 48 h in the presence of 0.01% DMSO (vehicle), 10 μM PQ, or 5 μM nitroquine (CI-679), and the infection rate of hepatocytes was assessed at the indicated time points. (**B**) Representative confocal images of *P. yoelii* EEFs in HepG2-CD81 cells treated with CI-679 and PQ at the indicated time points are shown. Scale bars represent 5 μm. (**C**) The inhibitory effect of CI-679 on the size of *P. yoelii* EEFs in HepG2-CD81 cells. (**D and F**) BALB/c mice were concurrently administered with a single dose of CI-679 (2 or 4 mg/kg, respectively, i.g., *n* = 5) at the time of 1,000 *P. yoelii* sporozoite challenge. Liver samples collected at 42 h post-infection were analyzed by qPCR for *P. yoelii* 18S rRNA levels relative to mouse GAPDH (mGAPDH) to measure parasite load (**D**). The protective efficacy was monitored in BALB/c mice (**F**). (**E and G**) C57BL/6 mice (*n* = 5) were concurrently administered with a single dose of CI-679 (2 or 4 mg/kg, respectively, i.g., *n* = 5) at the time of challenge with 10,000 *P. berghei-*luciferase sporozoites. Bioluminescence image indicating the intensity of luciferase (**E**, left), with total flux analysis over dosage (**E**, right). The protective efficacy was monitored in C57BL/6 mice (**G**). Three independent repeats have been performed for each experiment. The data are represented as the mean ± SD. One-way ANOVA (**A, C, D, and E**) or log-rank test (**F and G**) was used for statistical analysis.

CI-679 (2,4-diamino-6-[(3,4-dichlorobenzyl)-nitrosoamino]-quinazoline), an antimalarial drug that targets the blood stage by inhibiting parasites’ utilization of folic acid, is considered one of the treatments for malaria (16). In our previous study, we also found CI-679 could effectively suppress the development of artemisinin-resistant *P. falciparum* C580Y strain *in vitro* (17). However, its efficacy in suppressing the development of the liver stage has not been clearly established. To investigate whether CI-679 could suppress the development of the liver stage, *P. yoelii* BY265-RFP sporozoites were incubated with CD81-expressing HepG2 cells (HepG2-CD81) at various time points (12, 24, 36, and 48 h) in the presence or absence of CI-679, and the development of the liver stage was monitored. Although no significant difference in the infection rate of parasites was observed in HepG2-CD81 cells treated with or without CI-679 or PQ (Fig. 1A), the size of pre-erythrocytic forms (EEFs) began to decrease after 24 h of incubation regardless of whether treated with CI-679 or PQ (Fig. 1B and C). After 36 h of treatment, the EEFs exposed to a relatively low concentration of CI-679 (5 μM) were significantly smaller than those treated with 10 μM PQ (Fig. 1B and C), indicating that CI-679 suppresses the liver-stage development at a relatively late stage, with a higher capacity than that of PQ.

To confirm the inhibitory effect of CI-679 on malaria liver-stage development, two *in vivo* models, such as BALB/c mice infected with *P. yoelii* BY265 sporozoites and C57BL/6 mice infected with *P. berghei*-luciferase sporozoites, were used to investigate the effect of CI-679 on liver-stage development. In both models, a single concurrent administration of CI-679 at the time of sporozoite infection effectively reduced liver parasite burden in a dose-dependent manner (Fig. 1D and E). Notably, a single concurrent dose of 4 mg/kg CI-679 completely prevented the emergence of *P. yoelii* BY265 blood-stage parasites in BALB/c mice, and 20% of *P. berghei* blood-stage parasites in C57BL/6 mice (Fig. 1F and G). Taken together, these results demonstrate that CI-679 can inhibit malaria liver-stage development at a relatively late stage.

### Causal prophylaxis with CI-679 induces protective immunity against malaria dependent on CD8^+^ T-cell responses

**Fig 2:**
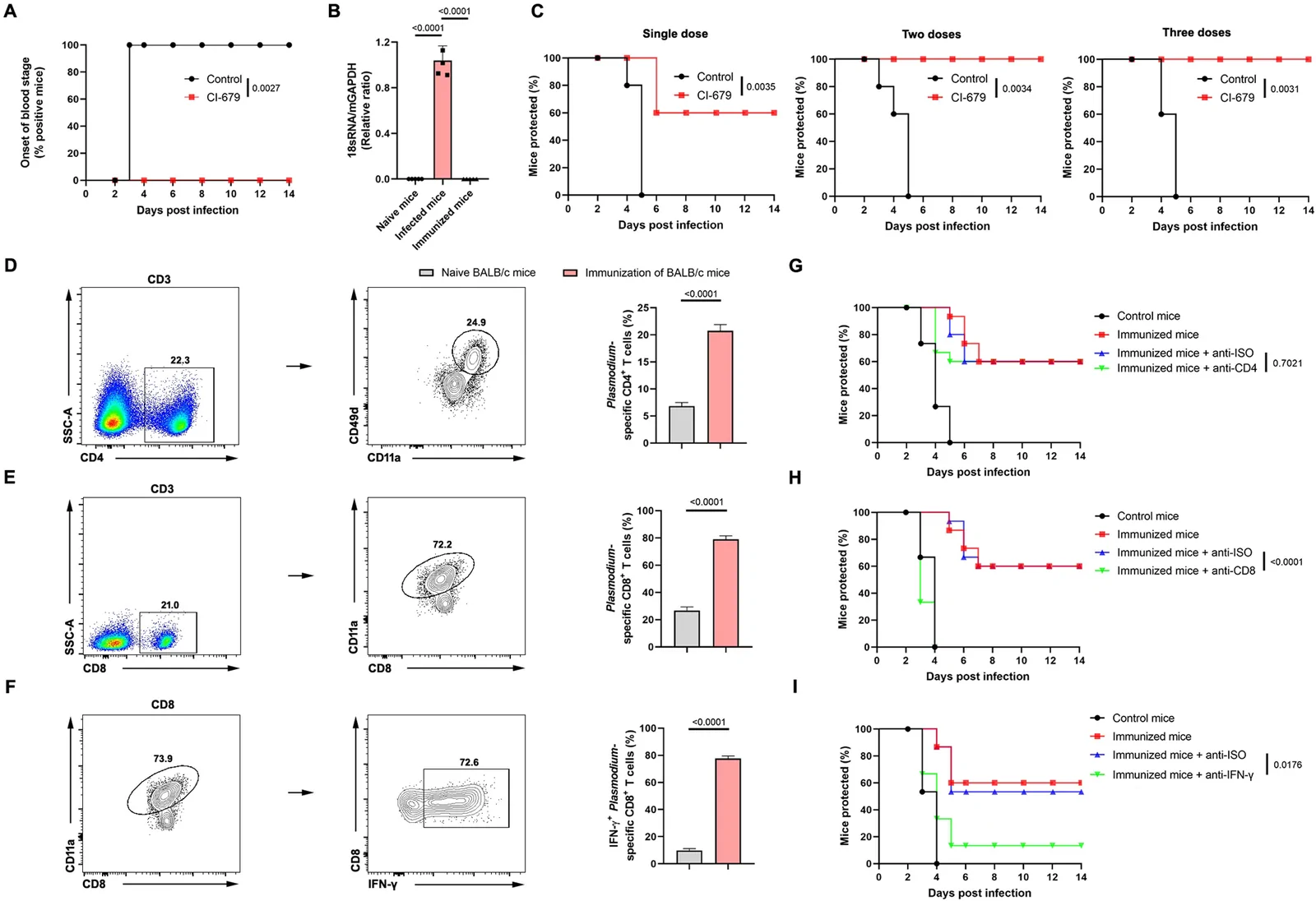
Causal prophylaxis with CI-679 induces CD8^+^ T-cell-dependent protective immunity against malaria. (**A**) The onset of blood-stage malaria parasites was monitored in BALB/c mice (*n* = 5) infected with 25,000 *P. yoelii* sporozoites, followed by treatment with CI-679 (10 mg/kg, i.g.) at 0, 1, and 2 days post-infection. (**B**) Blood samples collected at 7 days post-infection were analyzed by qPCR for *P. yoelii* 18S rRNA levels relative to mouse GAPDH (mGAPDH) to measure parasite load. (**C**) The protective efficacy of mice (*n* = 5) immunized with a single dose (left), two doses (middle), or three doses (right) of CI-679-attenuated sporozoites. (**D-F**) FACS analyses of liver *Plasmodium*-specific CD4^+^ T cells (**D**), CD8^+^ T cells (**E**), and IFN-γ^+^ *Plasmodium*-specific CD8^+^ T cells (**F**), and statistical analysis of the percentages from the naïve and immunized BALB/c mice (*n* = 5 for each group). (**G-I**) Protective efficacy of the pooled immunized mice (*n* = 15) depleted of CD4^+^ T cells (**G**), CD8^+^ T cells (**H**), or IFN-γ (**I**), followed by the challenge of *P. yoelii* sporozoites. The data are represented as the mean ± SD. One-way ANOVA (**B**), unpaired Student’s *t*-test (**D-F**), or log-rank test (**A**, **C**, **G-I**) was used for statistical analysis. Two to three times were performed for each experiment with similar results.

To evaluate whether causal prophylaxis with CI-679 attenuated sporozoites induces protective immunity against malaria, the sporozoite immunizing dose was increased from 10,000 to 25,000 to enhance the immunogenicity. Correspondingly, the dose of CI-679 was also increased from 4 to 10 mg/kg to prevent the onset of blood stage. As the half-life of CI-679 is only 27 h (18), CI-679 was administered at 0, 1, and 2 days after receiving *P. yoelii* sporozoites, to kill the blood stage parasite possible breakthrough from the liver stage. As a result, treatment with CI-679 in this schedule completely prevented the onset of blood stage (Fig. 2A), which was confirmed by quantitative PCR (qPCR) detection (Fig. 2B). This indicated CI-679 could effectively eliminate any parasites breakthrough from the liver stage, increasing the safety of the regimen. Remarkably, a single dose of CI-679-attenuated *P. yoelii* sporozoites provided approximately 60% protection against sporozoite challenge in the BALB/c mice, while two or three doses resulted in sterile protective immunity (Fig. 2C). These protective effects were not attributable to residual CI-679 following immunization with the attenuated sporozoite vaccine, as no protection against sporozoite challenge was observed in mice treated with CI-679 alone (Fig. S1A and B).

Notably, splenectomy prior to immunization with CI-679-attenuated *P. yoelii* sporozoites in BALB/c mice did not alter protective immunity, regardless of whether mice received one or three vaccine doses (Fig. S1C). We therefore mainly focused on liver lymphocyte immune dynamics post-immunization. The immunization with CI-679-attenuated sporozoites significantly activated *Plasmodium*-specific CD4^+^ and CD8^+^ T cells in the mouse liver, as indicated by an increased frequency of CD49d^hi^ CD11a^hi^ *Plasmodium*-specific CD4^+^ T cells and CD11a^hi^ CD8α^lo^ *Plasmodium*-specific CD8^+^ T cells (Fig. 2D and E and Fig. S2A and B). Additionally, the frequency of IFN-γ-secreting *Plasmodium*-specific CD8^+^ T cells was greatly elevated in mice immunized with CI-679-attenuated sporozoites (Fig. 2F). To determine whether CD4^+^ and CD8^+^ T cells were necessary for the protection conferred by the CI-679-attenuated sporozoites, either CD4^+^ T cells, CD8^+^ T cells, or IFN-γ were depleted prior to challenging the immunized mice. While the depletion of CD4^+^ T cells did not significantly affect the protective efficacy of the immunized mice (Fig. 2G), the depletion of CD8^+^ T cells or IFN-γ reduced the protection rate to 0% or 13%, respectively (Fig. 2H and I). These results demonstrate that the protection induced by CI-679-attenuated sporozoites primarily relies on parasite-specific CD8^+^ T-cell responses, consistent with previous studies on other chemo-attenuated sporozoites.

### Protective immunity induced by CI-679-attenuated vaccine varies with different host and parasite combinations

**Fig 3:**
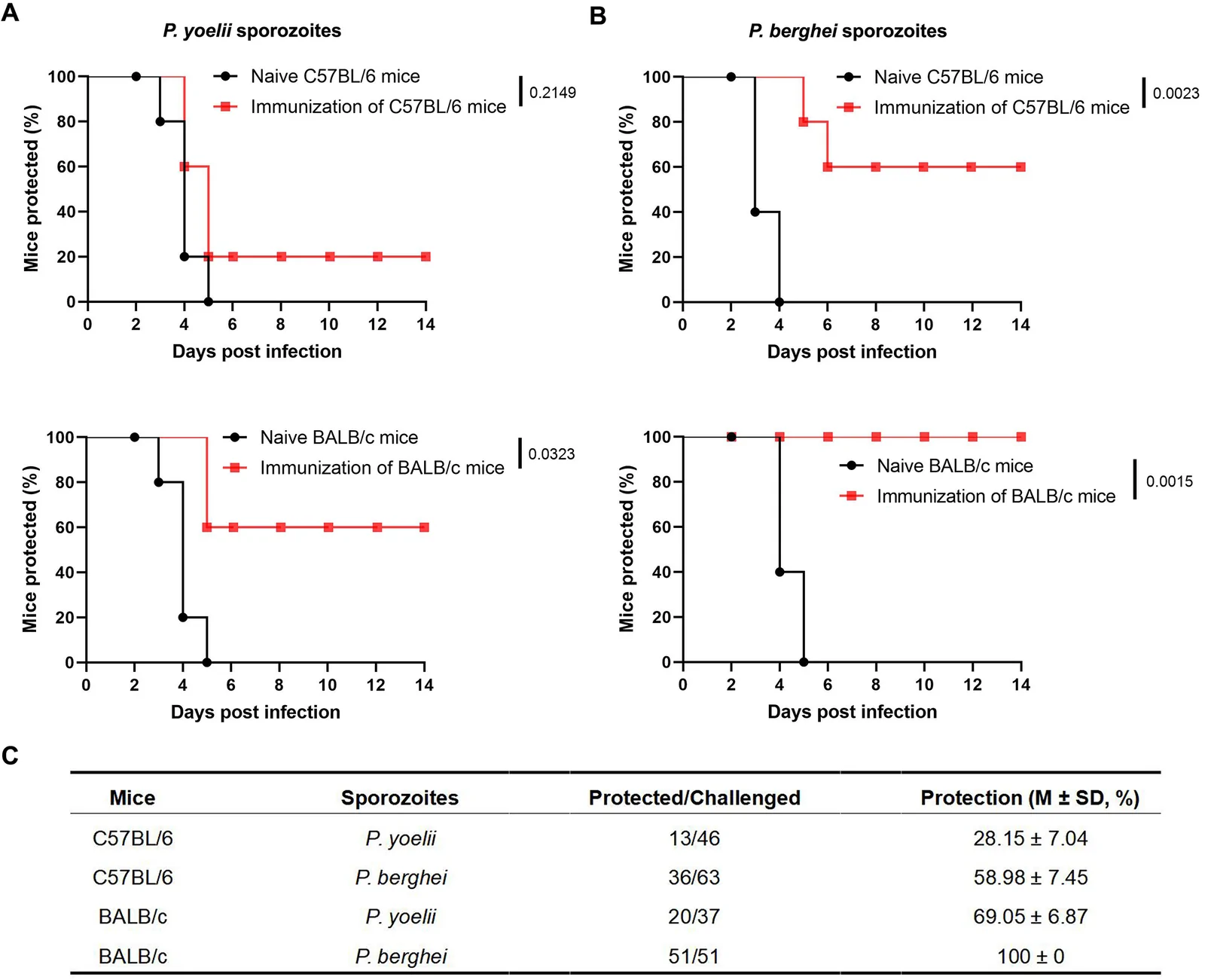
CI-679-attenuated vaccine induces variable protective immunity depending on host and parasite combinations. (**A**) Protective efficacy in C57BL/6 and BALB/c mice (*n* = 5) immunized with *P. yoelii* sporozoites against a challenge with 1,000 homologous sporozoites. (**B**) Protective efficacy in C57BL/6 and BALB/c mice (*n* = 5) immunized with *P. berghei* sporozoites against a challenge with 1,000 homologous sporozoites. All experiments were independently repeated at least three times, and representative results from one experiment are shown (**A and B**). (**C**) Summary of the protective efficacy in immunized mice following a challenge with 1,000 homologous sporozoites. The data are represented as the mean ± SD. The log-rank test was used for statistical analysis (**A and B**).

Next, the protective efficacy of C57BL/6 and BALB/c mice vaccinated with a single dose of CI-679-attenuated *P. yoelii* or *P. berghei* was compared. The efficacy of protection was evaluated by challenging the vaccinated mice with an intravenous (i.v.) injection of homologous viable sporozoites 21 days after immunization. A single dose of CI-679-attenuated *P. yoelii* sporozoites protected 28.15% ± 7.04% of C57BL/6 mice and 69.05% ± 6.87% of BALB/c mice against a challenge with 1,000 homologous sporozoites. In contrast, a single dose of CI-679-attenuated *P. berghei* sporozoites conferred protection to 58.98% ± 7.45% of C57BL/6 mice and 100% ± 0% of BALB/c mice against a challenge with 1,000 homologous sporozoites (Fig. 3A through C). Thus, the highest level of protection (100%) was achieved when BALB/c mice were immunized with attenuated *P. berghei* sporozoites; an intermediate protection rate (~60%) was achieved in BALB/c mice immunized with attenuated *P. yoelii* or C57BL/6 mice immunized with attenuated *P. berghei* sporozoites. Whereas C57BL/6 mice vaccinated with attenuated *P. yoelii* sporozoites showed only ~30% protection. These results are consistent with previous studies on irradiation-attenuated sporozoites (19).

### Different levels of anti-CSP antibodies do not contribute to the distinct protective immunity induced in two genetically distinct mouse strains

**Fig 4:**
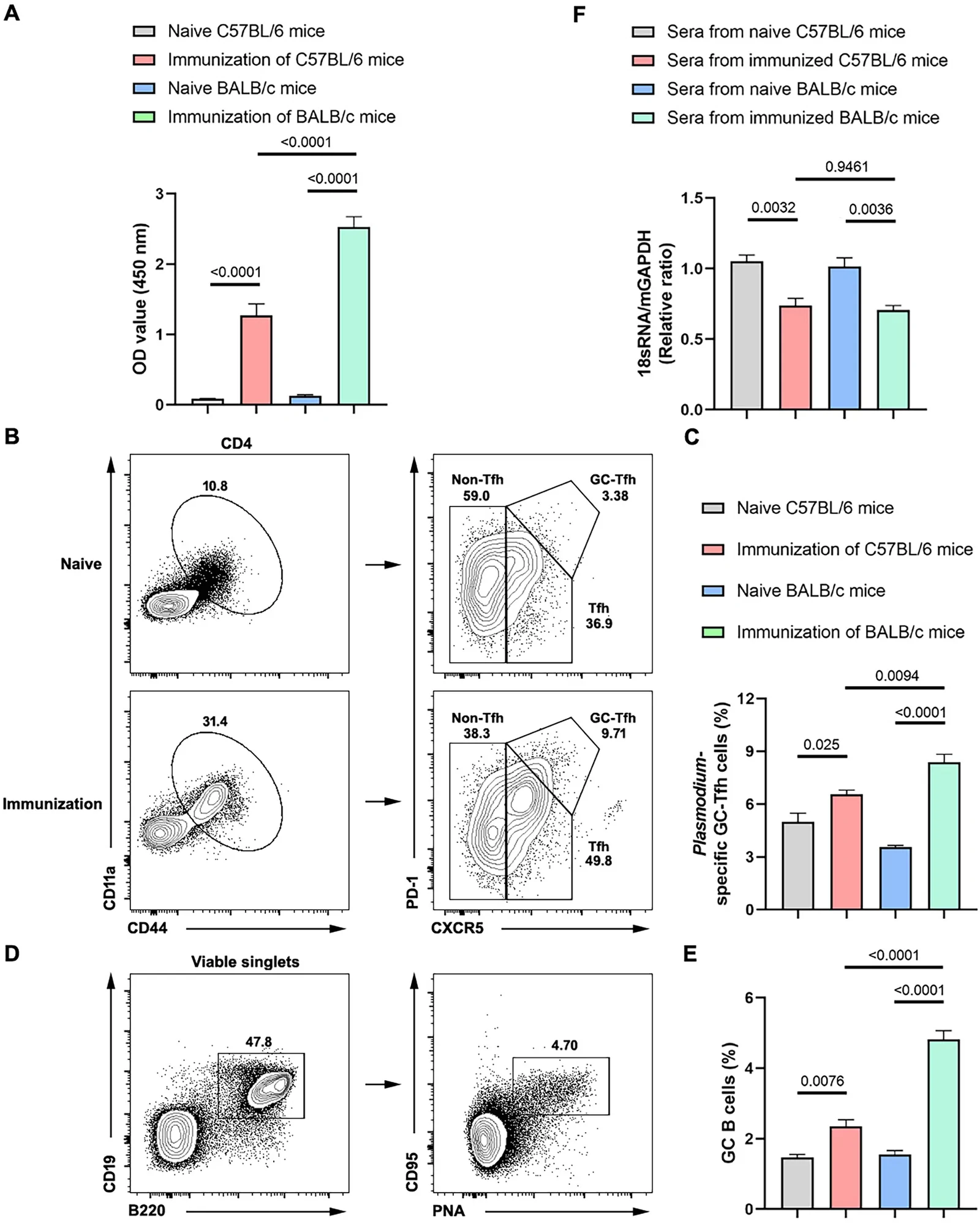
Anti-CSP antibody levels are unrelated to differential protection in genetically distinct mouse strains. (**A**) The anti-CSP antibody levels in the sera were compared between the vaccinated BALB/c and C57BL/6 mice. (**B and C**) Gating strategy (**B**) and frequency comparison (**C**) of splenic GC-Tfh cells (CD11a^+^CD44^+^PD-1ʰⁱCXCR5ʰⁱ) in immunized BALB/c and C57BL/6 mice. (**D and E**) Gating strategy (**D**) and frequency comparison (**E**) of splenic GC B cells (CD19^+^B220^+^CD95^+^PNA^+^) in immunized BALB/c and C57BL/6 mice. (**F**) Naïve mice were adoptively transferred with sera from immunized BALB/c or C57BL/6 mice, followed by *P. berghei* sporozoite challenge. Liver parasite burden was assessed by qPCR at 42 h post-infection. The data represent the mean ± SD. One-way ANOVA analysis (**A**, **C**, **E-G**) was used for statistical analysis. All experiments were repeated three times.

In addition to CD8^+^ T-cell responses, immunization with attenuated sporozoites also stimulated the production of antibodies against their surface proteins (20, 21). The circumsporozoite protein (CSP) is one of the key protective antigens in attenuated sporozoite vaccines (22), and high titers of anti-CSP antibodies can effectively prevent malaria infection either by the infected mosquito bite or i.v. sporozoite challenges (23). Therefore, we were interested in whether the differing levels of anti-CSP antibodies between C57BL/6 and BALB/c mice contribute to their varying degrees of protection. Immunization resulted in significantly higher titers of *P. berghei* CSP-specific antibodies in both C57BL/6 and BALB/c mice compared to naïve controls. Notably, the levels of CSP-specific antibodies were significantly greater in immunized BALB/c mice than in immunized C57BL/6 mice (Fig. 4A). As follicular helper T (Tfh) cells help prime germinal center (GC) B cells to initiate antibody responses against pathogens, we further investigated the roles of splenic Tfh cells and GC B cells induced by CI-679-attenuated sporozoites. The percentage of splenic PD-1^+^CXCR5^+^ GC-Tfh cells in BALB/c mice immunized with CI-679-attenuated *P. berghei* sporozoites was higher than those in immunized C57BL/6 mice (Fig. 4B and C). Similarly, the percentage of CD95^+^PNA^+^ GC B cells was elevated in both the *P. berghei*-C57BL/6 model and the *P. berghei*-BALB/c model. However, the percentage of CD95^+^PNA^+^ GC B cells in the *P. berghei*-BALB/c group was significantly higher than in the *P. berghei*-C57BL/6 group (Fig. 4D and E).

Next, we assessed the ability of sera collected from immunized C57BL/6 and BALB/c mice to neutralize sporozoites challenged through i.v. injection. No significant difference in liver parasite load was observed in naïve mice challenged with sporozoites after adoptive transfer of sera from either immunized C57BL/6 or BALB/c mice (Fig. 4F). It is important to note that the i.v. challenge with sporozoites will circumvent the inhibitory effects of anti-CSP antibodies on sporozoites in the skin (24). The role of varying levels of anti-CSP antibodies in the protective efficacy of these two mouse strains against mosquito bite challenges has yet to be clarified. Therefore, although immunized BALB/c mice produced higher levels of anti-CSP antibodies, in the context of intravenous sporozoite challenge, these differences do not account for the variability in protective efficacy observed between these two genetically distinct mouse strains.

### The distinct protective efficacy is attributed to varying levels of liver CD8^+^ Trm cells induced in two different mouse strains

To investigate the mechanisms underlying the differences in protective efficacy observed between immunized C57BL/6 and BALB/c mice, we conducted single-cell RNA sequencing (scRNA-seq) on lymphocytes isolated from the livers of both mouse strains at 3 weeks post-immunization. t-Distributed stochastic neighbor embedding (t-SNE) analysis categorized liver lymphocytes into eight clusters based on cell-specific molecular markers, including B cells (*Cd79a* and *Cd79b*), T cells (*Cd3e*), NK cells (*Ncr1*), plasma cells (*Jchain*), plasmacytoid dendritic cells (pDCs; *Siglech*), conventional dendritic cells (cDCs; *Itgax* and *H2-Ab1*), and monocytes (*Itgam* and *Lyz2*) (Fig. S3A and B and D). We observed significant differences in cell frequencies across nearly all clusters between the immunized C57BL/6 and BALB/c mice (Fig. S3C).

**Fig 5:**
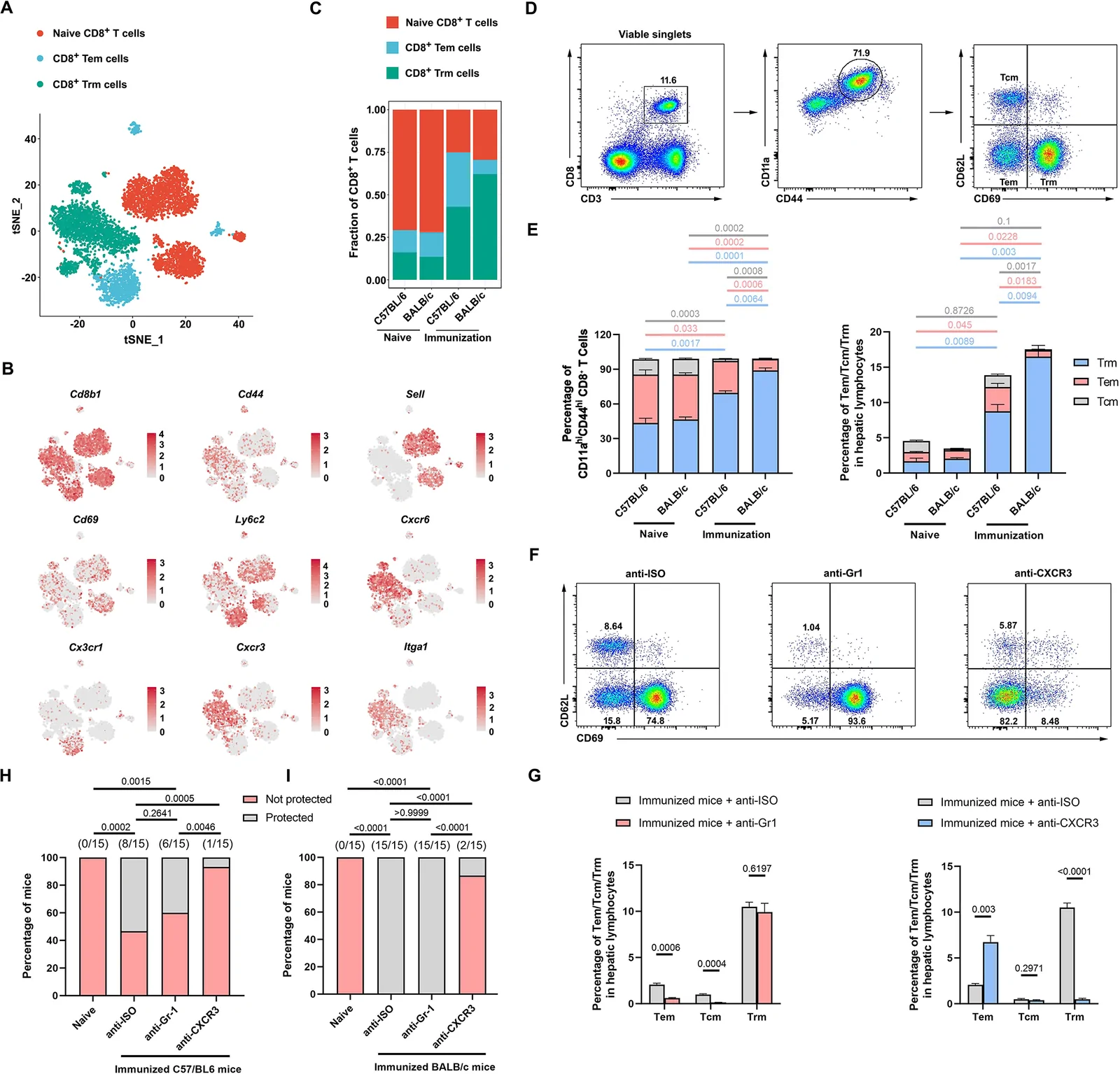
Differential protection linked to liver CD8^+^ Trm cell levels in two mouse strains. (**A and B**) Integrated scRNA-seq analysis of four experimental mouse groups: naïve C57BL/6, naïve BALB/c, immunized C57BL/6, and immunized BALB/c. scRNA-seq analysis on hepatic lymphocytes. t-SNE plots (**A**) and cell-specific molecular marker genes of hepatic lymphocytes (**B**). (**C**) Frequency comparison of Tem, Tcm, and Trm cells in the livers of CI-679-attenuated sporozoites immunized BALB/c and C57BL/6 mice. (**D and E**) Flow cytometry analyses of the percentage of hepatic CD8^+^ Tcm (CD62L^+^CD69^−^), Tem (CD62L^−^CD69^−^), and Trm (CD62L^−^CD69^+^) cells on day 21 after immunization with CI-679 attenuated sporozoites (**D**), and statistical analysis of the percentages of Tem, Tcm, or Trm cells among CD11a^hi^CD44^hi^CD8^+^ T cells or total lymphocytes from naïve and immunized BALB/c and C57BL/6 mice (**E**) (*n* = 5 for each group). (**F and G**) Representative flow cytometry plots illustrate Tem cell depletion (via anti-Gr-1 antibodies) or Trm cell depletion (via anti-CXCR3 antibodies) more than 21 days post-immunization (**F**), and comparison of the percentage of Tem, Tcm, and Trm cells between mice treated with specific depletion antibodies and those receiving the corresponding isotype control antibodies (**G**) (*n* = 3 for each group). (**H and I**) Percentages of C57BL/6 (**H**) or BALB/c mice (**I**) that were protected and not protected against a challenge with 1,000 *P. berghei* sporozoites, as measured by blood parasitemia up to day 14. Numbers above bars indicate proportions of protected to total mice. Data are pooled from three independent experiments (*n* = 5 for each group). The data represent the mean ± SD. One-way ANOVA (**E**, **H, and I**) or unpaired Student’s *t*-test (**G**) was used for statistical analysis. All experiments were performed once (**A-C**) or three times (**D-I**).

Previous studies have indicated that CD8^+^ T cells, especially effector memory CD8^+^ T cells (Tem) and tissue-resident CD8^+^ T cells (Trm), are the predominant protective immunity elicited by attenuated sporozoite vaccines (25–27). Therefore, T cells were extracted and further subdivided into CD4^+^ T cells (*Cd3e* and *Cd4*), CD8^+^ T cells (*Cd3e* and *Cd8b1*), NKT cells (*Cd3e* and *Ncr1*), and γδ T cells (*Cd3e* and *Tcrg-C2*) (Fig. S4A and B and D). Both immunized strains demonstrated significantly elevated CD8^+^ T-cell frequencies relative to non-immunized controls (Fig. S4C). Then, the CD8^+^ T cells were further divided into naïve CD8^+^ T cells, Tem cells, and Trm cells mainly based on the cell-specific molecular marker genes, including *Cd44*, *Sell*, *Cd69*, *Ly6c2*, *Cxcr6*, *Cx3cr1*, *Cxcr3*, *Itga1*, and *Klf2* (Fig. 5A and B and Fig. S5A and B). While the proportion of Tem cells was significantly higher in the livers of immunized C57BL/6 mice than that in those of BALB/c mice, the frequency of Trm cells was greater in BALB/c mice (Fig. 5C).

The differing frequencies of Tem and Trm cells in the livers of CI-679-attenuated sporozoite immunized C57BL/6 and BALB/c mice were confirmed by FACS analysis. CD8^+^ T cells were categorized into central memory CD8^+^ T cells (Tcm; CD62L^+^CD69^−^), Tem (CD62L^−^CD69^−^), and Trm cells (CD62L^−^CD69^+^), as previously described (Fig. 5D). Among these populations, Trm cells exhibited the most significant expansion, making up the majority of memory CD8^+^ T cells, while Tcm cells constituted the smallest proportion in both immunized C57BL/6 and BALB/c mice (Fig. 5E). Although the proportion of Tem cells in immunized BALB/c mice was lower than that in immunized C57BL/6 mice, the proportion of Trm cells in immunized BALB/c mice was significantly higher than in immunized C57BL/6 mice (Fig. 5E). To ensure that 3 weeks post-immunization is sufficient for accurately defining the real Trm cells, we compared their frequency in mice at 21 and 35 days post-immunization. Our findings indicated that extending the observation period from 21 to 35 days did not impact the frequency of liver Trm cells or the protective efficacy in the immunized mice (Fig. S6A and C). Consequently, for the following study, we selected the 21 days post-immunization time point to analyze both protective efficacy and Trm responses, thereby shortening the experimental period.

To determine whether the differences in Trm frequency between immunized C57BL/6 and BALB/c mice contribute to distinct protective efficacy, we selectively depleted circulating memory CD8^+^ T cells (anti-Gr1) and Trm cells (anti-CXCR3) in both strains more than 21 days post-immunization, as previously described (Fig. 5F and G) (26). Depletion of circulating memory CD8^+^ T cells with anti-Gr1 antibody had no statistically significant impact on protective efficacy in either mouse strain (Fig. 5H and I). In contrast, depleting Trm resulted in a substantial reduction in protective efficacy for both immunized C57BL/6 and BALB/c mice, lowering their protection levels to a similar extent (Fig. 5I). These findings indicate that the distinct protective efficacy is indeed linked to the varying levels of liver Trm cells induced in the two mouse strains.

### IL-15 significantly enhances Trm responses and protective efficacy of CI-679-attenuated vaccine-immunized C57BL/6 mice

**Fig 6:**
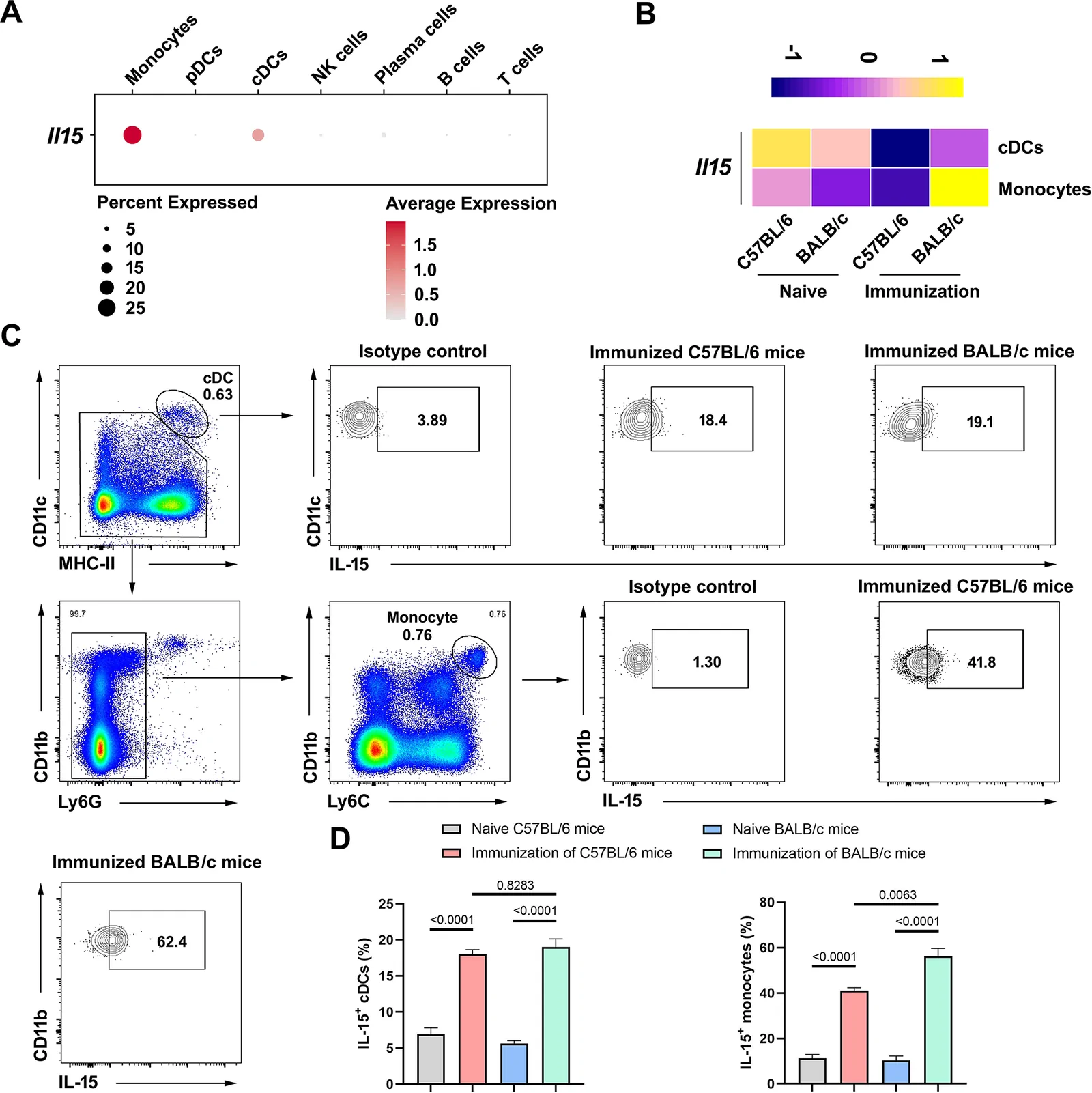
IL-15 level is elevated in monocytes from the livers of immunized BALB/c mice. (**A**) Integrated scRNA-seq analysis of four experimental mouse groups: naïve C57BL/6, naïve BALB/c, immunized C57BL/6, and immunized BALB/c. Dot plot of *Il15* gene expression in the indicated cells. (**B**) Heatmap showing the expression of *Il15* gene in hepatic cDCs and monocytes. (**C**) Representative FACS analyses of IL-15 expression on the surface of cDCs or monocytes from the immunized C57BL/6 or BALB/c mice. (**D**) Statistical analysis of the percentages of IL-15^+^ cDCs or IL-15^+^ monocytes from immunized C57BL/6 or BALB/c mice (*n* = 3 for each group). Unpaired Student’s *t*-test (**D**) was used for statistical analysis. Three independent experiments were performed with similar results for panel **C**.

**Fig 7:**
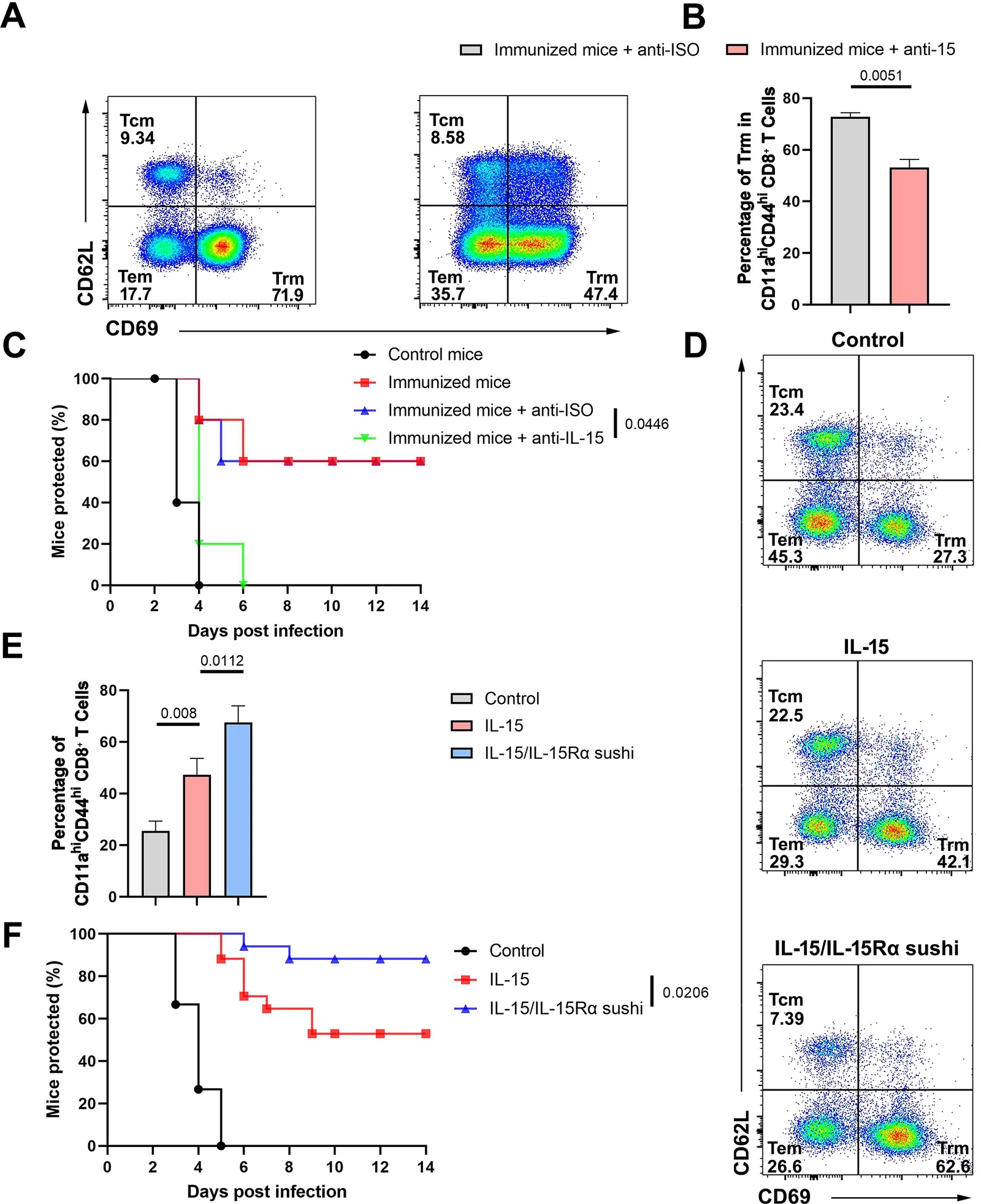
IL-15 significantly enhances Trm responses and protective efficacy of CI-679-attenuated vaccine-immunized C57BL/6 mice. (**A and B**) Representative FACS plots (**A**) and statistical analysis (**B**) of hepatic Trm cells from immunized mice treated with anti-isotype or anti-IL-15 antibodies. (**C**) Protective efficacy of the immunized C57BL/6 mice treated with anti-isotype or anti-IL-15 antibodies following the challenge with 1,000 *P. berghei* sporozoites (*n* = 5 for each group). (**D and E**) Representative FACS plots (**D**) and FACS analysis of the frequency (**E**) of liver Trm in the CI-679-attenuated sporozoite vaccine immunized mice (*n* = 3) supplemented with or without IL-15 sushi complex. (**F**) Protective efficacy of the CI-679-attenuated sporozoite vaccine immunized mice co-administered with or without IL-15 sushi complex, data pooled from three independent experiments. The data represent the mean ± SD (**B and E**). Unpaired Student’s *t*-test (**B**) and one-way ANOVA (**E**) were used for statistical analysis; the Log-rank test was applied for panels **C** and **F**. Three independent experiments were performed with similar results.

The formation of liver Trm cells has been reported to be regulated by local inflammatory cytokines, such as IFN-α and IL-15 (28, 29). Next, the expression of those cytokines in myeloid cells of the immunized C57BL/6 and BALB/c mice was analyzed. We observed that IL-15 was predominantly expressed in cDCs and monocytes (Fig. 6A). Notably, only IL-15 expression in monocytes was found to be significantly higher in the livers of the immunized BALB/c than in those of C57BL/6 mice (Fig. 6B). This finding was further confirmed by FACS analysis, which revealed comparable IL-15 expression levels in liver cDCs between immunized C57BL/6 and BALB/c mice, and demonstrated elevated IL-15 levels in monocytes from the livers of immunized BALB/c mice (Fig. 6C and D). Following the neutralization of IL-15 by anti-IL-15 antibody, the frequency of parasite-specific CD8^+^ Trm cells was significantly reduced (Fig. 7A and B), resulting in a decrease in protection for C57BL/6 mice immunized with CI-679-attenuated sporozoites to levels comparable to those of the control mice (Fig. 7C). Thus, our results demonstrate that the distinct protective efficacy in two mouse strains is associated with the expression level of IL-15, which enhances Trm generation in the immunized C57BL/6 mice.

It is well established that IL-15 secreted by DCs or macrophages/monocytes is trans-presented to CD8^+^ T cells by IL-15 Rα, which binds to the IL-2R/IL-15R β and γ heterodimer (30). Due to the short half-life of soluble IL-15 *in vivo*, the relatively stable IL-15/IL-15Rα sushi domain complex is commonly used to activate CD8^+^ T cells (31, 32). To evaluate whether IL-15 could enhance the protective efficacy of SPZ-CVac, we prepared the IL-15/IL-15Rα sushi domain complex (IL-15/IL-15Rα sushi) and co-administered it with CI-679-attenuated sporozoite vaccines. The frequencies of Trm and the protective efficacy of the immunized mice were then assessed (Fig. 7D and E). Compared to the administration of IL-15 alone, the concentration of IL-15 in the IL-15/IL-15Rα sushi treated group was significantly higher over time (Fig. S7). Furthermore, supplementation with the IL-15/IL-15Rα sushi not only significantly increased liver Trm frequency (Fig. 7E), but also improved the protective efficacy of the immunized C57BL/6 mice (Fig. 7F). Taken together, these data suggest that IL-15 can significantly enhance the protective efficacy of CI-679-attenuated sporozoite vaccines, possibly by increasing liver Trm frequency.

## DISCUSSION

SPZ-CVac is considered one of the promising attenuated sporozoite vaccines due to its robust immunogenicity. However, there are concerns regarding the safety of antimalarial drugs that target either the liver or blood stages. In this study, we identified the antimalarial drug CI-679, which is typically used to eliminate malaria blood stages, as an effective agent for arresting the development of late EEFs. A single administration of CI-679 as causal prophylaxis significantly prevented the onset of the blood stage and conferred considerable protection against sporozoite challenge. Additionally, we observed that the protective efficacy induced by the CI-679-attenuated sporozoite vaccine varied among different host-parasite combinations, with high protection being dependent on the frequency of hepatic CD8^+^ Trm cells mediated by IL-15. Co-administration of IL-15/IL-15Rα sushi significantly elevated the protective efficacy of CI-679-attenuated sporozoite vaccine in C57BL/6 mice. Therefore, we successfully established a novel SPZ-CVac with enhanced safety and immunogenicity via CI-679, demonstrating the essential role of IL-15-mediated CD8^+^ Trm responses in the high protection induced by the CI-679-attenuated sporozoite vaccine.

The use of antimalarial drugs targeting liver stages is a well-established strategy for preventing malaria infection. Primaquine, a key member of the 8-aminoquinoline class, effectively prevents *P. falciparum* infections and the relapse of *P. vivax* and *P. ovale* (33, 34). However, its short half-life and the risk of severe hemolysis in patients with glucose-6-phosphate dehydrogenase (G6PD) deficiency limit its use (35). Tafenoquine, another 8-aminoquinoline, has a longer half-life of 14 days, but G6PD testing is still necessary before administration (36, 37). Alternatively, antifolate drugs have been employed to prevent malaria in epidemic regions (38). Sulfadoxine-pyrimethamine, which inhibits dihydropteroate synthase, is widely used in malaria-endemic areas (39). Additionally, causal prophylaxis with pyrimethamine effectively attenuates live sporozoites and induces both homologous and heterologous sterile immunity against *P. falciparum* challenges (8). In this study, we identified another class of antifolates, CI-679, which effectively arrested EEFs development at the late stage, and causal prophylaxis with CI-679 also primes protective immunity against subsequent sporozoite challenges. Compared to other SPZ-CVac, the CI-679-attenuated sporozoite vaccine has a potentially favorable safety rationale because of its dual liver- and blood-stage activity. However, findings derived from rodent malaria models cannot be directly extrapolated to human malaria. To date, no clinical data have demonstrated that CI-679 attenuates *P. falciparum* liver-stage parasites in humans (40). Further toxicological assessments, evaluation of resistance risks in human malaria, and head-to-head comparisons with other clinical SPZ-CVac regimens are required to validate the translational potential of this vaccination strategy. Resolving these knowledge gaps will be critical for determining whether this SPZ-CVac approach can progress toward clinical development for human malaria prophylaxis.

Consistent with the essential role of liver CD8^+^ Trm cells in the protective immunity conferred by irradiation-attenuated sporozoite vaccines (26), the protection elicited by our CI-679-attenuated sporozoite vaccine was also dependent on liver CD8^+^ Trm cell responses. Additionally, we observed that the protective efficacy of the CI-679-attenuated sporozoite vaccine varied among different host-parasite combinations, consistent with findings from a previous study (41). In that study, the differences in protective efficacy were attributed to both the *Plasmodium* species and strain-specific background genes in non-hematopoietic cells, though the exact mechanisms remain unclear (41). In the present study, we found that, following intravenous sporozoite challenge, the variation in protection between BALB/c and C57BL/6 mice against the same *Plasmodium* strain after vaccination with CI-679-attenuated sporozoites correlated with differences in the frequencies of hepatic Trm cells. An additional important consideration is the route of sporozoite challenge used in this study. All challenge experiments were performed via intravenous sporozoite injection, which bypasses the skin stage of natural mosquito-borne infection and may underestimate the functional contribution of anti-CSP antibodies. Accordingly, our conclusion that antibody responses do not drive differential vaccine protection is limited specifically to the i.v. challenge setting. Since we lack experimental data from mosquito-bite challenge, this represents a notable limitation of the current work. Future investigations employing mosquito bite challenge will be required to assess whether humoral immunity plays a more prominent protective role under physiologically relevant transmission conditions.

Elucidating the mechanisms underlying the formation of liver CD8^+^ Trm cells could offer crucial insights for designing highly effective pre-erythrocytic vaccines (9). While local antigen presentation is not a prerequisite for the conversion of primed CD8^+^ T cells into CD8^+^ Trm cells in the liver, it can enhance their generation. In contrast, a local inflammatory environment is essential for this transformation (28). Studies have demonstrated that IFN-γ and IL-15 promote the generation of hepatic CD8^+^ Trm cells (28), whereas type I IFN exerts a suppressive effect (29). Among these cytokines, IL-15 is deemed the most critical for driving the differentiation of CD8^+^ Trm cells (28). Consistently, we also demonstrated the essential role of IL-15-mediated Trm responses in the high protection induced by the CI-679-attenuated sporozoite vaccine. However, we could not formally conclude that monocyte-derived IL-15 is the essential source supporting Trm development, as global IL-15 blockade inhibits IL-15 from all cellular compartments. Verification of monocytes as the functionally relevant IL-15 source will require monocyte-specific *Il15* conditional knockout mice, which we propose as future investigations. Notably, co-administration of IL-15/IL-15Rα sushi complex significantly enhanced hepatic Trm responses and protection in C57BL/6 mice immunized with CI-679-attenuated vaccine, indicating the potential adjuvant role of IL-15 in enhancing the efficacy of pre-erythrocytic stage vaccines. The requirement for IL-15 at the effector phase of sporozoite-induced protection is conceptually reminiscent of earlier studies establishing IL-12 as a critical mediator of pre-erythrocytic immunity (42–44). However, these two cytokines serve distinct functions. IL-12 drives the initial priming of IFN-γ-producing T cells that underpin early protection. In contrast, our data demonstrate that IL-15 acts at a later, effector-memory stage to support liver-resident Trm cell maintenance and function. Thus, IL-12 and IL-15 represent complementary, non-redundant cytokine axes that together shape durable liver-stage immunity following the attenuated sporozoite vaccination. Recently, arresting malaria parasites at the late liver stage via genetic deletion of the *Mei2* and *LINUP* genes, or treatment with the antimalarial compounds WM382 and MK-7602, confers markedly improved protective efficacy (45, 46). These findings highlight a viable strategy for boosting the immunogenicity of attenuated SPZ vaccines. Here, we propose an alternative strategy employing IL-15 as an adjuvant to augment the protective potency of attenuated SPZ vaccines.

IL-15 receptors (IL-15R and IL-15αβγ) are expressed on various subsets of CD8^+^ T cells, including effector (Teff), Tem, Tcm, and Trm cells (47, 48). IL-15 has been shown to play a pivotal role in promoting the survival of effector CD8^+^ T cells and the generation of memory CD8^+^ T cells (47, 49). However, it remains unclear whether IL-15 enhances Trm generation by acting on the IL-15R of Teff, Tem, or Tcm cells or by promoting Trm proliferation through direct action on its own IL-15R (50). Additionally, the mechanisms by which SPZ-CVac induces IL-15 expression are still not yet fully understood. In this study, we found that IL-15 was predominantly expressed by cDCs and monocytes. Notably, there were differences in IL-15 expression levels in monocytes between immunized BALB/c and C57BL/6 mice, which was closely associated with the frequency of CD8^+^ Trm cells in these mice. Although IL-15Rα of macrophages has been shown to trans-present IL-15 to promote the differentiation of memory CD8^+^ T cells (51), whether monocyte-derived IL-15 promotes the differentiation of CD8^+^ Trm cells through trans-presentation by IL-15Rα remains to be determined in future research by using monocyte-specific *Il15* conditional knockout mice. Finally, future research should also focus on elucidating the mechanism by which CI-679-attenuated *P. berghei* sporozoites induce higher levels of IL-15 production in monocytes from BALB/c mice compared to those from C57BL/6 mice.

In summary, we demonstrated that antifolates CI-679 effectively inhibit the development of EEFs, and a single prophylactic dose of CI-679 can induce strong protective immunity against subsequent sporozoite challenge. Furthermore, we revealed that the high protection induced by the CI-679-attenuated sporozoite vaccine was dependent on the IL-15-mediated Trm responses, and the supplement of IL-15/IL-15Rα sushi complex could significantly elevate the protection induced by the CI-679-attenuated vaccine. Therefore, we not only established a novel SPZ-CVac with potentially favorable safety, but also provide mechanistic evidence supporting IL-15 as an adjuvant to enhance the efficacy of pre-erythrocytic stage vaccines.

## MATERIALS AND METHODS

### Mice and parasites

*Plasmodium yoelii* BY265, *P. yoelii* BY265-RFP, and *P. berghei* ANKA were maintained in our laboratory. Specific pathogen-free BALB/c and C57BL/6 female mice at the age of 6–8 weeks were purchased from GemPharmatech Corporation Limited (Chengdu, China). Kunming mice were purchased from the Laboratory Animal Center of the Army Medical University (Chongqing, China). Mice were infected with parasites via i.p. injection of 1 × 10^5^ parasitized red blood cells (pRBCs).

### Mosquitoes rearing and infection

*Anopheles stephensi* (Hori strain, *An. stephensi*) in our laboratory was maintained at 27°C at a relative humidity of 70–80%, and fed with 10% sugar solution containing 0.5% para-aminobenzoic acid. After being infected with parasites for 3 days, mice were fed to 3- to 6-day-old female *An. stephensi* starved for 24 h. After feeding for 30 min, engorged mosquitoes were removed and maintained at 19–20°C for *P. berghei* or 23–24°C for *P. yoelii*, under a 12-h light/dark cycle. Seven days after the blood meal, mosquito midguts were dissected, and oocyst numbers were counted using a light microscope to determine the infection level.

### Sporozoite isolation and challenge

Mosquito salivary glands were dissected and collected at 17–19 days and 19–21 days post-infection with *P. yoelii* and *P. berghei*, respectively, in RPMI 1640 medium supplemented with 2.5 mg/mL amphotericin B (Sangon Biotech, Shanghai, China), 100 units/mL penicillin, and 100 μg/mL streptomycin (Beyotime, Beijing, China). After grinding in Eppendorf tubes and centrifugation, salivary gland sporozoites were collected and counted. For the *in vitro* assay, sporozoites were incubated with HepG2-CD81 cells at a ratio of 1:3. Three hours after incubation, the supernatant was discarded and replaced with fresh culture medium with agents as described above. For the *in vivo* assay, mice were challenged with an i.v. injection of 1,000 sporozoites unless otherwise stated.

### Effect of CI-679 on *Plasmodium* liver-stage development

BALB/c mice were administered i.p. with CI-679 at a dose of 2 mg/kg or intragastrically (i.g.) at doses of 2, 4, and 8 mg/kg daily for three consecutive days, followed by a challenge with 1,000 *P. yoelii* sporozoites. To evaluate the time-dependent effect of CI-679 on *Plasmodium* liver-stage development, BALB/c mice were challenged with 1,000 *P. yoelii* sporozoites or 10,000 *P. berghei* sporozoites, and subsequently treated with a single intragastric administration of 4 mg/kg of CI-679 at −3, −2, −1, 0, 1, and 2 days post-challenge, respectively. The onset of blood stage was monitored daily using Giemsa staining for 2 weeks.

### *In vitro* effect of CI-679 on EEFs development

HepG2-CD81 cells were seeded at a density of 1 × 10^6^ cells per well on the slide in 24-well plates followed by addition of 5 × 10^5^ *P. yoelii* BY265-RFP sporozoites. Then, cells were incubated in the presence of 5 μM CI-679 or 10 μM PQ. At 12, 24, 36, and 48 h post-incubation, cells were fixed with 4% paraformaldehyde. Parasites were visualized with DAPI to stain the nuclei. Confocal images were obtained with an LSM800 confocal system (Zeiss) equipped with visible and ultraviolet laser lines. The infection rate of EEFs, as well as the size of the EEFs in HepG2/CD81 cells at the indicated time points, was measured.

### Immunization and challenge

Mice were i.v. injected with 25,000 *P. berghei* or *P. yoelii* sporozoites, followed by administration of primaquine diphosphate (PQ, 60 mg/kg, i.p.) or CI-679 (10 mg/kg, i.g.). The booster immunization dosages were given at a 2-week interval. In each experiment, untreated age-matched control mice were injected with equivalent numbers of sporozoites to confirm the infectivity of the sporozoites used for immunization. Three weeks after the last immunization, mice were challenged with 1,000 sporozoites via the tail vein. The onset of blood-stage malaria parasites was monitored by Giemsa-stained thin blood smears to determine the protective efficacy for 2 weeks.

### Bioluminescence measurement

Forty-two hours after sporozoite challenge, the challenged mice were injected with D-luciferin (100 mg/kg) and anesthetized. After mice were immobilized, they were then placed in an IVIS Spectrum imager to evaluate bioluminescence by measuring the radiance for 5 min from the abdomen.

### *In vivo* depletion assay

To deplete CD4^+^ or CD8^+^ T cells, immunized mice were i.p. injected with 200 μg of either anti-CD4 (clone GK1.5; BioXCell), anti-CD8a (clone YTS 169.4; BioXCell), or the corresponding isotype control IgG 1 day prior to and on the day of the challenge. To deplete IFN-γ, immunized mice were i.p. injected with 200 μg of either anti-IFN-γ (clone XMG1.2; BioXCell) or rat IgG1, κ isotype control (clone HRPN; BioXCell) antibodies 1 day prior to and on the day of the challenge. To deplete circulating CD8^+^ T cells, immunized mice were i.p. injected with two doses (250 μg and then 50 μg) of either anti-Gr-1 (clone NIMP-R14; BioXCell) or IgG2b, κ isotype-control (clone LTF-2; BioXCell) antibodies on days 22 and 26 after immunization, respectively (7 and 3 days before the challenge with 1,000 live *P. berghei* sporozoites). To deplete Trm cells, immunized mice were i.v. injected with two doses (200 μg and then 100 μg) of either anti-CXCR3 (clone CXCR3-173; BioXCell) or Armenian Hamster IgG isotype control (eBio299Arm, eBioscience) antibodies on days 26 and 28 after immunization, respectively (3 and 1 days before challenge with 1,000 live *P. berghei* sporozoites). To neutralize IL-15, immunized mice were i.p. injected with 200 μg of either anti-mouse IL-15 (clone AIO.3, BioXCell) or IgG2b, λ isotype control (clone LTF-2; BioXCell) antibodies from days 8 to 20, with a 1-day interval between each injection, after immunization, followed by challenge with 1,000 live *P. berghei* sporozoites on day 21.

### Assessment of sera neutralization

To investigate the sera neutralization of sporozoites *in vivo*, serum samples from immunized mice were collected 21 days after immunization, naïve mice were i.v. injected with 200 μL sera once daily for 3 days. Then, the serum-transferred mice were challenged with 1,000 sporozoites. Forty-two hours later, liver parasite burden was detected by qPCR.

### Preparation of intrahepatic lymphocytes

At 7 or 21 days after immunization, livers of the immunized mice were perfused with PBS through the hepatic portal vein. Livers were mechanically disrupted using the plunger of a 5-mL syringe and strained through a 70-μm nylon filter. The cell suspension was collected into a 50-mL conical tube and centrifuged at 300 × *g* for 10 min at 4°C without braking. The cell pellet was resuspended in 30 mL ice-cold serum-free RPMI 1640 and centrifuged at 30 × *g* for 3 min at 4°C without braking. The supernatants were collected and transferred to another 50 mL conical tube and centrifuged at 300 × *g* for 10 min. The cell pellet was resuspended in 3 mL 33% (vol/vol) Percoll; 3 mL 70% (vol/vol) Percoll was added to the lower layer; and then centrifuged at room temperature at 750 × *g* for 20 min without brake. White precipitate at the interface was collected, and ice-cold serum-free RPMI 1640 was added to replenish the volume to 10 mL, and then centrifuged at 500 × *g* at 4°C for 10 min. The final cell pellet containing hepatic lymphocytes was resuspended in 1 mL red blood cell lysis buffer for 2–3 min, quenched with 4 mL MACS buffer (PBS, 1 mM EDTA, and 0.5% FBS), and then centrifuged at 500 × *g* at 4°C for 3 min.

### Flow cytometry analysis

The hepatic lymphocytes were resuspended in 1 mL RPMI 1640, centrifuged at 300 × *g* for 10 min. After cell counting, 2 × 10^6^ lymphocytes were collected and treated with anti-CD16/32 (clone 93; BioLegend) for 30 min to block Fc receptors. In the cell-surface staining assay, cells were stained with specific antibodies for 45 min. For intracellular staining for IFN-γ, cells were fixed, permeabilized (Cytofix/Cytoperm reagent; BD Biosciences), and stained with anti-mouse IFN-γ (clone XMG1.2; BioLegend). For membrane IL-15 staining, the cells were labeled with biotin-conjugated anti-IL-15 mAb (PeproTech) at 4°C for 30 min, followed by the addition of streptavidin-allophycocyanin (BD Pharmingen). To analyze the frequency of GC Tfh and GC B cells in the spleen of the immunized mice, splenocytes were prepared as previously described (52). After Fc receptors were blocked as mentioned above, cells were stained with specific antibodies to detect both GC Tfh and GC B cells.

The antibodies included: anti-mouse CD3-PE-Cy7 (clone 17A2; BioLegend), anti-mouse CD4-APC (clone GK1.5; BioLegend), CD4-FITC (clone RM4-5; BioLegend), CD8α-APC-Cy7 (clone 53-6.7; BioLegend), CD8α-PerCP-Cy5.5 (clone 53-6.7; BioLegend), CD11a-PerCP-Cy5.5 (clone M17/4; BioLegend), CD11a-PE (clone 2D7; BD Biosciences), CD11a-FITC (clone M17/4; eBioscience), CD44-PE-Cy7 (clone IM7; BioLegend), CD44-APC-Cy7 (clone IM7; BioLegend), CD69-APC (clone H1.2F3; BD), CD69-PE (clone H1.2F3; BioLegend), CD62L-FITC (clone MEL14-H2.100; Miltenyi), CD62L-PE (clone MEL14; eBioscience), CD11c-APC (clone N418; BioLegend), MHC-II-FITC (clone M5/114.15.2; BioLegend), CD11b-Percp-Cy5.5 (clone M1/70; BioLegend), Ly6G-BV510 (clone 1A8; BioLegend), CD19-PerCP-Cy5.5 (clone 6D5; BioLegend), CD95-PE (clone 15A7; eBioscience), rat anti-mouse CXCR5 (clone 2G8; BD Pharmingen), PD-1-PE (clone 29F.1A12; BioLegend), PNA (cat. no. FL1071, Vector Laboratories), and B220-APC (clone RA3-6B2; BioLegend). Unless indicated differently, all FACS antibodies were applied at a final dilution of 1:100.

Dead cells were excluded by using the LIVE/DEAD Fixable Dead Cell Stain Kit following the manufacturer’s protocol (cat. no. L34994, Invitrogen). All data were collected using a BD FACS Canto II cytometer (BD Biosciences) or a BD FACS Celesta cytometer (BD Biosciences) and analyzed using FlowJo v10 software (Tree Star Inc.).

### Splenectomy

Adult mice were anesthetized through the i.p. injection of chloral hydrate (Sangon Biotech). The abdominal wall was shaved prior to making a 1-cm incision; then, the spleen was freed from the surrounding tissue by blunt dissection. Blood vessels were ligated using 5–0 silk suture, and the skin incision was closed with 4–0 silk suture. Control animals were sham splenectomized on the same SPZ-CVac. This surgery was completed 2 weeks before the immunization. To minimize variability, all surgeries were performed by the same individual.

### Enzyme-linked immunosorbent assay

Three weeks post-immunization, serum samples were collected from the mice, and the antibody titer against the *P. berghei* ANKA CSP epitope was determined after 1:1,000 dilution. The *P. berghei* ANKA CSP epitope, containing three repeats (PAPPNANA-PAPPNANA-PAPPNANA), was synthesized by Wuhan GeneCreate Biological Engineering Co., Ltd. (Wuhan, China) and purified by high-performance liquid chromatography (HPLC) using a Waters XBridge C18 column (4.6 × 250 mm × 5 mm; Waters Corporation) on a Waters Corporation Prominence HPLC System (MA, USA). For the detection of CSP-specific antibodies, Nunc MaxiSorp immunoplates (Nalge Nunc, USA) were coated overnight at 4°C with peptides at a concentration of 1 μg/mL in 100 μL per well. Subsequently, the plates were incubated with serial dilutions of sera obtained from both control and immunized mice. Following a washing step with washing buffer, the plates were further incubated with horseradish peroxidase (HRP)-conjugated anti-mouse IgG. Then, 3,3′,5,5′-tetramethylbenzidine (TMB) substrate (BioLegend) was added. Finally, the absorbance at 450 nm was measured using a spectrophotometer.

### Single-cell RNA sequencing

Three weeks after the final immunization with CI-679-attenuated *P. berghei* sporozoites, hepatic lymphocytes from the immunized BALB/c and C57BL/6 mice were prepared as described above and subsequently submitted for single-cell RNA sequencing in accordance with the manufacturer’s instructions for the Chromium Single Cell 3′ Reagent Kits v3 (10× Genomics). Cellular suspensions (1 × 10^3^ cells/µL) were loaded onto the Chromium Controller (10× Genomics) to generate Gel Bead-In-Emulsions (GEMs). Barcoded sequencing libraries were prepared according to the manufacturer’s protocol for the Chromium Single Cell 3′ Reagent Kits v3. Sequencing was conducted on a NovaSeq 6000 (Illumina, San Diego, CA, USA) with paired-end sequencing of 150 nucleotides per end using one lane per sample. Raw sequencing data were processed using Cell Ranger (10× Genomics, v6.0.1), with the “count” function to align raw reads with the STAR aligner against the mm10 reference genome. Library preparation and sequencing were performed at Jiayin Biotechnology Ltd. (Shanghai, China). Data analysis, including unsupervised clustering, was conducted using the Seurat package (v4.4.0) in R (v4.3.2). t-SNE was employed for dimensionality reduction in this study. General cell types were annotated based on cell-type-specific marker-gene expression and the genes driving cluster heterogeneity. The Seurat package was also used for differential gene expression analysis to compare expression profiles between the two groups. Heatmaps were generated using R (v4.3.2) with the pheatmap package (v1.0.12). The volcano plot was created using R (v4.3.2) and the Enhanced Volcano package (v1.18.0). The RAW data have been deposited in the GEO database with accession number GSE305696.

### Quantitative PCR

The liver tissues of immunized mice were harvested and homogenized. The total RNA was isolated using TRIzol (Invitrogen) according to the manufacturer’s instructions. Total RNA was then reverse-transcribed to cDNA using PrimeScript RT reagent Kit with gDNA Eraser (TAKARA). *Plasmodium yoelii* 18S rRNA and *P. berghei* 18S rRNA were determined by HiScript III All-in-one RT SuperMix Perfect for qPCR (Vazyme) on a CFX Connect Real-Time System (Bio-Rad). Relative quantitative results were normalized to the mouse GAPDH gene, which was used as the internal control. Specific primers targeting the 18S rRNA or mouse GAPDH were as follows: *P. berghei* 18S rRNA forward primer, 5′-GGTTTTATAATTGGAATGATGGGAAT-3′; *P. berghei* 18S rRNA reverse primer, 5′-ACGCTATTGGAGCTGGAATTACC-3′; *P. berghei* 18S rRNA Probe, 5′-FAM-AACCTTCCCAAAAAT-MGB-3′; *P. yoelii* 18S rRNA forward primer, 5′-CTTGGCTCCGCCTCGATAT-3′; *P. yoelii* 18S rRNA reverse primer, 5′-TCAAAGTAACGAGAGCCCAATG-3′; *P. yoelii* 18S rRNA Probe, 5′-FAM-CTGGCCCTTTGAGAGCCCACTGATT-TAMRA-3′; mouse GAPDH forward primer, 5′-GCACAGTCAAGGCCGAGAA-3′; mouse GAPDH reverse primer, 5′-CCTCACCCCATTTGATGTTAGTG-3′; and mouse GAPDH Probe, 5′-FAM-TTCTCCAGGAGCGA GAC-MGB-3′.

### Preparation of IL-15 sushi complex and evaluation of its adjuvant effect on CI-679-attenuated vaccines

The IL-15 sushi complex was prepared as previously described (53). The cDNA sequence encoding mouse IL-15, linked to the sushi domain of mouse IL-15α (amino acids 1–77), via the linker sequence SSGGGSGGGGSGGGGSGGGGSGGGS, was synthesized and cloned into the pcDNA3.4 vector. The resulting recombinant plasmids were then transfected into CHO cells, and the fusion protein (IL-15/IL-15Rα sushi domain) was purified using AmMag Ni Magnetic Beads, which specifically target the His tag at the C-terminus of the fusion protein. To evaluate its adjuvant effect on CI-679-attenuated vaccines, 10 μg of the IL-15 sushi complex was co-administered i.m. with CI-679-attenuated sporozoite vaccines three times at 2, 48, and 96 h post-vaccination. Three weeks after immunization, the frequency of hepatic Trm cells was determined, and the protective efficacy of the mice was recorded following challenge with live sporozoites.

### Statistical analysis

All statistical analyses were performed with Prism software (version 9.0, GraphPad Inc.). Data are shown as the mean ± SD unless otherwise stated. Statistically significant differences between the two groups were determined using the unpaired Student’s *t*-test, one-way ANOVA, or two-way ANOVA, and *P* < 0.05 was considered statistically significant.

## Data Availability

All data needed to evaluate the conclusions in the paper are present in the paper and/or the supplemental material. All unique/stable reagents generated in this study are available from the corresponding author upon reasonable request. The single-cell RNA sequencing data generated in this study have been deposited in the GEO database (https://www.ncbi.nlm.nih.gov/geo/) under accession number GSE305696.

## SUPPLEMENTAL MATERIAL

Supplemental figures mbio.01131-26-s0001.docx Figures S1 to S7.

ASM does not own the copyrights to Supplemental Material that may be linked to, or accessed through, an article. The authors have granted ASM a non-exclusive, world-wide license to publish the Supplemental Material files. Please contact the corresponding author directly for reuse.
