# Supplementary material for: The high protection of a novel chemo-attenuated sporozoite vaccine requires IL-15-mediated liver CD8+ tissue-resident memory T-cell responses
Source: mBio. 2026 Sep 24;17(10):e01131-26. doi: 10.1128/mbio.01131-26 (PMC13712793; doi:10.1128/mbio.01131-26)
Supplement: Supplementary file 1 [file mbio.01131-26-s0001.docx]

**Supplementary Information**

**The high protection of a novel chemo-attenuated sporozoite vaccine requires IL-15-mediated liver CD8^+^ tissue-resident memory T cell responses**

Nie Tan,^a^ Chengyu Zhu,^a,c^ Teyang Li,^a^ Shubin Su,^a^ Shiming Jiao,^a^ Yuanli Gao,^a^ Yan Ding,^a^ Taiping Liu,^a,b^ ^#^ & Wenyue Xu,^a,b^ ^#^

^a^Department of Pathogenic Biology, Army Medical University (Third Military Medical University), Chongqing, 400038, China.

^b^Key Laboratory of Extreme Environmental Medicine, Ministry of Education of China, Chongqing, 400038, China.

^c^Department of Clinical Laboratory, People’s Hospital of Deyang City, Deyang, 618000, China

Running Head: IL-15-mediated liver CD8^+^ Trm response via SPZ-CVac

#Address correspondence to Wenyue Xu, [xuwenyue@tmmu.edu.cn](mailto:xuwenyue@tmmu.edu.cn) or Taiping Liu, [liutaiping@tmmu.edu.cn](mailto:liutaiping@tmmu.edu.cn)

Nie Tan, Chengyu Zhu and Teyang Li contributed equally to this work. Author order was determined on the basis of seniority.

**Supplementary Fig. 1 | Evaluation of the effect of residual CI-679 or splenectomy on protective efficacy in BALB/c mice immunized with attenuated *P. yoelii* sporozoites. A, B** BALB/c mice (n = 13) were challenged with 25,000 *P. yoelii* sporozoites, followed by treatment with CI-679 (10 mg/kg, *i.g.*) at 0, 1 and 2 days post-infection, or received an equivalent dose of CI-679 alone. Thirty-five days later, the mice were challenged with 1000 sporozoites. At 42 hours post-challenge, hepatic parasite burden was determined by qPCR (**A**, n=3). Protective efficacy was evaluated (**B**, n=10). **C** Protective efficacy in BALB/c mice undergoing splenectomy (SP) or sham splenectomy (SSP) prior to immunization with CI-679-attenuated *P. yoelii* sporozoites. n = 5 for each group. Experiment was repeated two times. The data are represented as mean±SD. One-way ANOVA (**A**) or log-rank test (**B, C**) was used for statistical analysis.

**Supplementary Fig. 2 | Full gating strategy of *Plasmodium*-specific CD4^+^ and CD8^+^ T cells. A** The gate strategy of FACS analyses of liver *Plasmodium*-specific CD4^+^ T cells (CD3^+^CD4^+^ CD49d^hi^CD11a^hi^). **B** The gate strategy of FACS analyses of liver *Plasmodium*-specific CD8^+^ T cells (CD3^+^CD8α^+^CD11a^hi^CD8α^lo^).

**Supplementary Fig. 3 | scRNA-seq analysis of murine hepatic lymphocytes. A** Unsupervised clustering was conducted on the t-SNE to discern clusters of cells exhibiting similar gene expression patterns. **B** The relative fractions of immune cell populations among all hepatic lymphocytes. **C** t-SNE visualization depicting the expression of canonical immune cell markers across each cell cluster of hepatic lymphocytes. **D** Violin plots show the expression levels of representative marker genes within each cell cluster of hepatic lymphocytes.

**Supplementary Fig. 4 | scRNA-seq analysis of murine hepatic T lymphocytes. A** Unsupervised clustering was conducted on the t-SNE to discern clusters of cells exhibiting similar gene expression patterns. **B** The relative fractions of immune cell populations among all hepatic T lymphocytes. **C** t-SNE visualization depicting the expression of canonical immune cell markers across each cell cluster of hepatic T lymphocytes. **D** Violin plots show the expression levels of representative marker genes within each cell cluster of hepatic T lymphocytes.

**Supplementary Fig. 5 | Canonical marker genes of memory CD8^+^ T cells. A** t-SNE visualization depicting the expression of canonical immune cell markers across each cell cluster of hepatic CD8^+^ T cells. **B** Violin plots show the expression levels of representative marker genes within each cell cluster hepatic CD8^+^ T cells.

**Supplementary Fig. 6 | Assessment of the frequency of Trm cells and the protective efficacy in mice at 21 days and 35 days post-immunization.** C57BL/6 mice were immunized with 25,000 CI-679 attenuated sporozoites, at 21 days and 35 days post-immunization, the mice were challenge with 1,000 sporozoites. **A, B** Flow cytometry analyses of percentage of hepatic CD8^+^ Tcm (CD62L^+^CD69^-^), Tem (CD62L^-^CD69^-^), and Trm (CD62L^-^CD69^+^) cells (**A**), and statistical analysis of the percentages of Trm cells from the the live C57BL/6 mice (**B**). n = 3 for each group. **C** Protective efficacy of immunized C57BL/6 mice challenged at indicated time post-immunization. n = 5 for each group. Three independent repeats have been performed for each experiment. The data are represented as mean±SD. One-way ANOVA (**B**) or log-rank test (**C**) was used for statistical analysis.

**Supplementary Fig. 7 | *In vitro* expression profile of IL-15 and IL-15/IL-15Rα sushi complex 24 hours post-transfection.** HEK293 cells were seeded in 6-well plates to 90%~95% confluence. For each well, 5 μg purified IL-15 mRNA or IL-15/IL-15Rα sushi mRNA was transfected. After 24 h, the concentration of IL-15 in the supernatant was quantified by enzyme-linked immunosorbent assay (ELISA). One-way ANOVA was used for statistical analysis.
